# Enhancing the knowledge and understanding domain of physical literacy: instrument validation and a Kahoot-supported intervention in physical education

**DOI:** 10.3389/fspor.2026.1799871

**Published:** 2026-03-27

**Authors:** Bogdan Sorin Olaru, Teodora Mihaela Iconomescu, Laurentiu-Gabriel Talaghir

**Affiliations:** Department of Individual Sports and Kinetotherapy, Faculty of Physical Education and Sport, “Dunarea de Jos” University of Galati, Galati, Romania

**Keywords:** Kahoot, knowledge and understanding, methodology development, PE teachers, physical education, physical literacy, validation

## Abstract

**Introduction:**

The Knowledge and Understanding (K&U) domain plays a fundamental role in Physical Literacy (PL), yet Physical Education (PE) lessons in Romania—and many Eastern European countries—remain largely sport-based, with limited emphasis on K&U. To support PL-oriented instructional practices, validated tools are needed to assess students' knowledge, as well as pedagogical approaches integrating digital learning methods. This study aimed (1) to develop and validate a standardized instrument for assessing the K&U domain of PL in PE, and (2) to examine the effectiveness of a year-long Kahoot-supported intervention designed to enhance this domain.

**Methods:**

The instrument was developed through a multi-stage validation process, including item generation, expert evaluation, pretesting, student interviews, and test–retest reliability assessment. Intervention effectiveness was examined using a mixed-methods design. The quantitative component employed a quasi-experimental design with an experimental group (*n* = 27) and a control group (*n* = 27). The experimental group participated in 14 Kahoot-supported theoretical lessons integrated into the PE curriculum, while the control group followed traditional lessons. A qualitative focus-group interview was conducted with the experimental group after the intervention, and data were analyzed using thematic analysis.

**Results:**

The instrument demonstrated high clarity, relevance, balanced difficulty, and excellent reliability (*r* = 0.94). Significant improvements were observed in the experimental group's K&U scores [*t*(25) = −8.66, *p* < 0.001; *d* = 1.7], while the control group showed no change [*t*(20) = −0.48, *p* = 0.63]. Post-test comparisons also favored the experimental group [*t*(45) = −4.75, *p* < 0.001; *d* = 1.39]. Qualitative thematic analysis revealed four themes: increased enthusiasm, positive competitiveness, facilitated learning, and suggestions for diversification.

**Discussion:**

The findings indicate that theoretical learning does not occur spontaneously during traditional sport-based PE lessons. Structured theoretical instruction, especially when supported by gamified digital tools, can enhance students' K&U and aligns with the holistic philosophy of PL.

**Conclusion:**

This study introduces the first standardized knowledge assessment instrument for Romanian PE and demonstrates the effectiveness and feasibility of Kahoot-supported theoretical lessons. This approach offers a practical pathway for countries transitioning from sport-based PE toward PL-oriented practices.

## 1 Introduction

The concept of Physical Literacy (PL) has been receiving increasing attention from researchers, policymakers, and practitioners across the education, sport, and public health sectors (1–4). The term is also explicitly referenced by the World Health Organization in its Global Action Plan on Physical Activity 2018–2030 (5), and UNESCO emphasizes that PL represents a key element of every Physical Education (PE) lesson (6).

At the global level, there is no consensus regarding the definition of PL. This lack of unity is generally accepted, as PL is considered to be culturally context-dependent (7). Therefore, from the outset we specify that in this paper we refer to one of the most widely used definitions of PL—namely, the one proposed by the International Physical Literacy Association—which states that PL can be described as “the motivation, confidence, physical competence, knowledge and understanding to value and take responsibility for engagement in physical activities for life” (8).

A particularly influential model conceptualizes PL as comprising four interconnected domains: affective (motivation and confidence), physical (physical competence), cognitive (knowledge and understanding), and behavioral (engagement in physical activities for life) (9–11). Among these, the Knowledge and Understanding (K&U) domain represents the cognitive component and is the primary focus of the present study. The K&U domain is considered a core element of the cognitive domain and a key attribute of PL. It refers to the acquisition of knowledge related to movement and health, encompassing individuals' ability to identify and articulate the essential qualities that influence the effectiveness of their own movement performance, as well as their understanding of the principles of embodied health, including fundamental aspects such as physical activity, sleep, and nutrition (12, 13).

In addition, recent literature has also employed the term “cognitive activation” to describe cognitively oriented teaching PE. In a systematic review, Töpfer et al. (14) conceptualize cognitive activation as an umbrella term encompassing several established pedagogical approaches, such as “Teaching Games for Understanding”, “concept-based physical education”, and “Sport Education”, all of which aim to stimulate students' in-depth mental engagement with sport- and movement-related learning content.

Cale and Harris (12) argue that the K&U domain is fundamental because it catalyzes the development of the other domains. This view is not at all incompatible with the holistic nature of PL. Naturally, all domains contribute to progress along the PL journey; however, individuals must know what to do, how to do it, and where—in other words, they require knowledge. Lundvall (15) similarly emphasizes that without acquiring knowledge, “part of the literacy ambition will be lost,” and young people “may not master the keys to lifelong learning through movement” (p. 117).

There is a bidirectional relationship between the K&U domain and the other domains. The K&U domain is enriched as the other domains develop—namely motivation, confidence, physical competence, and social interaction. However, the relationship also operates in the opposite direction: through the acquisition of knowledge, individuals gain the theoretical support needed to understand how they can further develop the other domains (13).

Considering the fundamental role of the K&U domain, the question naturally arises regarding both the methods through which this knowledge should be conveyed and the need to monitor its acquisition—that is, assessment. In a systematic review paper, Iconomescu et al. (16) highlighted various intervention designs aimed at implementing health-related knowledge across different educational levels. The most notable outcomes were observed when the intervention took place in schools, underscoring the central role of the educational setting in fostering knowledge acquisition.

In line with this, Corbin et al. (17) regard the PE lesson as one of the most powerful tools available for implementing PL, as it provides a unique opportunity for all children to acquire knowledge, build understanding, and develop healthy habits.

The specialized literature identifies several approaches to implementing and assessing knowledge within PE lessons (14, 16). For instance, The Society of Health and Physical Educators (SHAPE America) articulates Standard 5 of the National PE Standards, stating that “a physically literate individual recognizes the value of physical activity for health, enjoyment, challenge, self-expression and/or social interaction” [(18), p. 4]. In the United States, the Conceptual Physical Education model offers a blended approach, combining classroom-based theoretical lessons with practical sessions conducted in the gym or on the field (19, 20).

In Australia, the curriculum for Years 11 and 12 includes “Physical Education Studies” (21), a program grounded in the idea that PE involves three forms of education: (1) education “in movement” (through sensory experience), (2) education “through movement” (addressing physical, emotional, social, and intellectual objectives), and (3) education “about movement”, which seeks to develop a rational understanding of physical activity by emphasizing anatomical, biomechanical, physiological, sociological, psychological, and philosophical knowledge (22).

For a long period, the PE lesson was perceived as one focused almost exclusively on practical content. As a result, its educational value was often underestimated—leading to situations in which PE held a lower status compared to other school subjects, even among teachers (23). Today, however, with the growing popularity of the PL concept and the fundamental role attributed to the K&U domain, PE can reaffirm its position as a key discipline within individuals' general education.

With these considerations in mind, translating the concept into practice requires research efforts aimed at identifying the most effective pedagogical methods through which the PE lesson can contribute to achieving PL objectives in general, and to developing the K&U domain in particular (12).

To date, the implementation of theoretical knowledge within PE lessons has predominantly relied on cognitively activating teaching practices, such as the use of open-ended tasks, structured reflection on sport-related experiences, social interaction, and scaffolding, all of which aim to stimulate students' in-depth mental engagement with learning content (14). While these approaches are well aligned with constructivist pedagogy, delivering theoretical knowledge in an effective and engaging manner within PE remains challenging.

One pedagogical strategy that may address this challenge is the use of digital game-based learning platforms such as Kahoot. Kahoot has been widely recognized as an effective educational tool that enhances student engagement, motivation, and knowledge acquisition across various academic subjects by incorporating elements of gamification, immediate feedback, and active participation (24–26). These features are particularly relevant to the K&U domain of PL, as they facilitate cognitive engagement and support the consolidation of theoretical concepts related to movement, health, and physical activity. Although Kahoot has been extensively studied in classroom-based subjects, its application within PE remains limited, especially in relation to supporting the development of PL. There is a lack of empirical research examining the effectiveness of Kahoot as a structured pedagogical tool for enhancing the K&U domain within the context of school-based PE. Addressing this gap is essential for identifying effective strategies to integrate theoretical learning into PE and support the broader implementation of PL-oriented practices.

Considering, on the one hand, the central role of the K&U domain in the implementation of PL, and on the other hand, the use of Kahoot as an effective teaching tool, the purpose of this paper is to examine the effectiveness of a year-long school intervention in which Kahoot is integrated into PE lessons to enhance the K&U domain. A secondary aim of the study is to develop a valid instrument for measuring the level of theoretical knowledge in PE among 5th-grade students in Romania. The following sections outline the methodological approach, present the results of the intervention, and discuss their implications for PE practice and future research.

## 2 Methods

This study employed a mixed-methods design consisting of two sequential phases. The first phase involved the development and validation of an instrument to assess the K&U domain of PL among 5th-grade students, based on the content of the official Romanian PE textbook.

The second phase consisted of a quasi-experimental intervention conducted over one school year with a convenience sample of 54 students, divided into an experimental group (*n* = 27) and a control group (*n* = 27). The experimental group participated in Kahoot-supported theoretical lessons integrated into PE classes, while the control group followed the traditional practical instruction. Both groups were assessed before and after the intervention. A post-intervention focus group interview was also conducted with the experimental group to explore students' perceptions of the learning experience.

The following subsections describe the procedures for instrument development and validation, intervention implementation, and qualitative data collection.

### 2.1 Instrument development

The development and validation of the instrument were guided by the principles of Classical Test Theory (CTT), a widely recognized framework for constructing and evaluating educational and psychological measurement instruments (27, 28). Within this framework, the quality of an assessment instrument is established through systematic procedures that ensure content validity, clarity, appropriate item difficulty, and measurement reliability. CTT emphasizes the importance of expert-based item development, pilot testing, and reliability analysis to produce consistent and interpretable scores that reflect the underlying construct being measured.

In accordance with these principles, the development process also followed established methodological guidelines for questionnaire design and validation (29–31), including item generation, expert evaluation, pretesting, cognitive interviews, and test–retest reliability assessment.

Given the purpose of our instrument—to measure the K&U domain of PL within the context of PE—we aimed to validate a questionnaire consisting of 25 items, each accompanied by six response options. A total of 25 items with six response options each was chosen based on two main considerations: the need to remain within the maximum attention span that students can dedicate to a test (48), and the examples provided by previous studies with similar objectives, which employed instruments of comparable length (49, 50). Regarding the response options, one of the six choices was always “I don't know”. The inclusion of this option was intended to prevent falsely recording correct or incorrect answers in situations where respondents might otherwise guess due to not knowing the correct response.

After establishing these general characteristics of the evaluation tool, the validation process unfolded across seven steps: (1) identifying thematic areas and distributing items; (2) generating the item pool; (3) selecting items; (4) pretesting; (5) conducting interviews; (6) performing the reliability test; and (7) developing a guide for the application of the instrument.

#### 2.1.1 Identifying thematic areas and distributing items

As mentioned above, the body of knowledge we aimed to assess corresponds to the PE textbooks available to Romanian PE teachers at the 5th-grade level for delivering theoretical content (32–34). To identify the factors of our instrument, we used the chapter structure of these textbooks, which follows the six main areas of the national curriculum: (1) Organization of Motor Activities; (2) Harmonious Physical Development; (3) Motor Skills; (4) Sports Disciplines; (5) Hygiene and Individual Protection; and (6) Behaviors and Attitudes.

After defining these thematic areas (factors), the 25 items were distributed across them based on the proportion of textbook content allocated to each area. In other words, the greater the amount of content (calculated as the percentage of pages) assigned to a thematic area, the more items from the total pool of 25 were allotted to that area.

#### 2.1.2 Generation of the item pool

In this stage, the three authors of the present paper (O.B.S., T.L.G., I.T.M.) examined the textbook content and independently generated, for each thematic area, ten times more items than the number established. For example, in the previous stage it was determined that the thematic area “Organization of Motor Activities” would ultimately include 4 items out of the total 25. Consequently, for this thematic area, each author was required to generate at least 40 items (4 * 10).

Each item consisted of a question accompanied by six response options, one of which had to be I don't know. Among the remaining five options, at least one—and at most five—had to be correct, while the others were incorrect.

#### 2.1.3 Item selection

In the third stage, three Romanian PE teachers working in secondary education—with 10, 12, and 16 years of professional experience—received the items generated in the previous stage and were asked to evaluate them across four dimensions: (1) the clarity of the question; (2) the clarity of the correct answer options; (3) the difficulty level of the item relative to a 5th-grade student, considering both the question and the answer options; and (4) the relevance of the concept assessed in the item (i.e., how important the specialist believes it is for a 5th-grade student to know the answer). Each dimension was rated using a 6-point Likert scale, with scores ranging from 0 to 5 (see Scoring Grid in the Supplementary Materials).

The purpose of having the items evaluated across four dimensions by three independent reviewers was to identify the 25 top-performing items. However, before selecting these items, Cronbach's alpha was calculated for the four scoring dimensions to test the internal consistency of the evaluators' ratings. Our target was to obtain *α* > 0.8. If this threshold was not met, items showing large discrepancies among reviewers were removed.

After achieving excellent internal consistency in the specialists' evaluations, the process continued by examining three performance criteria for each item. As will be shown, these criteria formed the basis for selecting the best-performing items: (1) Item Clarity Score; (2) Item Relevance Score; and (3) Item Difficulty Score.

*Item Clarity Score* was calculated by summing the values assigned by the three evaluators for Dimension 1 (clarity of the question) and adding the sum of the values they assigned for Dimension 2 (clarity of the correct answer options). The maximum possible score for this criterion was 30 points (5 points * 3 evaluators + 5 points * 3 evaluators). Items with a clarity score below 25 points were eliminated. In this way, we ensured that less clear items were filtered out and that the subsequent stages of the process proceeded only with the items demonstrating the highest level of clarity.

*Item Relevance Score* was obtained by summing the values assigned by the three evaluators for Dimension 4 (how important the specialist believes it is for a 5th-grade student to know the answer to that item). The maximum possible score for this criterion was 15 points (5 points * 3 evaluators). The remaining items from the previous stage were grouped into their respective thematic areas (the factors), and only those with a relevance score above 13 points were retained. The rationale behind this step was to select items that assessed the most important and meaningful concepts, after having already filtered out items with insufficient clarity.

*Item Difficulty Score* was calculated by summing the values assigned by the three evaluators for Dimension 3 (the difficulty level of the item relative to a 5th-grade student). The maximum possible score for this criterion was 15 points (5 points * 3 evaluators). The remaining items from the previous stages were then sorted within their respective thematic areas according to their difficulty score. Items for each thematic area were selected based on the criterion that 50% should be of low difficulty and 50% of high difficulty. This approach ensured a balanced distribution of difficulty levels. In cases where an odd number of items was required for a thematic area, one medium-difficulty item was selected first, and the remaining items were divided according to the principle described above.

After completing these steps, version 1.0 of the instrument for measuring theoretical knowledge levels was developed.

#### 2.1.4 Pretesting

In this stage, the 25 items selected previously were tested on a sample of 30 students (6th grade). Since the objective was to examine the characteristics of the items rather than those of the respondents, there was no need to address the issue of sample representativeness. This stage had two objectives: (1) identifying any issues related to test administration, and (2) classifying respondents based on their scores.

Based on the pretesting results, 12 students were selected (High–Low: top 20% and bottom 20%) to participate in the next stage—the interview phase.

#### 2.1.5 Interview

In this stage, the 12 students selected from the pretesting phase were invited to participate in a round of interviews. Using a semi-structured interview guide, the objective was to identify any ambiguities related to the understanding of the items and their correct answer options (see Scoring Grid in the Supplementary Materials). To assist students and encourage clarification, three supporting questions were used: “Is there any word you don’t know?”, “What does the word ‘X’ mean?”, and “What do you understand when reading this question?”.

#### 2.1.6 Reliability test

The test–retest method (35) was used to determine whether the final instrument, comprising the 25 rigorously selected items, provides predictable and repeatable results. For this purpose, the same sample (30 subjects, distinct from those used in the pretesting stage) completed the instrument twice, with a 21-day interval between administrations. A paired *t*-test was then applied to the test–retest scores.

#### 2.1.7 Guide for instrument application

In this stage, a guide for test administration was developed to standardize the conditions under which subjects are evaluated. The guide ensures consistent testing procedures by providing standardized training and instructions for the personnel administering the test.

The guide consists of three components: (1) an overview describing the context, objectives, and steps involved in the test administration process; (2) a standardized instruction script to be read to the participants prior to testing, ensuring objectivity and uniformity; and (3) instructions for collecting and interpreting the results.

### 2.2 The intervention

In this study, an educational intervention was implemented over the duration of an entire school year, with the aim of examining how the integration of structured theoretical lessons supported by gamified digital tools (the Kahoot platform) may influence learning in the context of PE classes. A convenience sample of 54 5th-grade students from a public school in Galați, Romania, was used. Participants were naturally distributed into a control group (*n* = 27) and an experimental group (*n* = 27), based on the school's existing organization of students into two distinct classes. Both classes were taught by the same PE teacher, allowing for control over variables related to teaching style and instructional expertise. The intervention took place between September 5, 2022, and June 16, 2023.

Students in the control group participated in PE lessons delivered in a traditional, fully practical format, consistent with standard PE instruction in Romanian schools. In this setting, theoretical information was conveyed informally during practical activities conducted either in the sports hall or on the field. In contrast, students in the experimental group took part in a total of 14 theoretical lessons delivered in the classroom throughout the school year (out of 76 total lessons per year), completely separate from the physical activity component. These lessons aimed to systematically teach the theoretical content specified in the curriculum using a mixed pedagogical structure. Specifically, during the first approximately 15 min of each lesson, the teacher delivered the content in a expository manner, followed by a consolidation phase implemented through a gamified methodology using Kahoot to enhance engagement and strengthen concept understanding.

The structure of the intervention was aligned with the Romanian national PE curriculum, which organizes instruction into learning units typically comprising 4 to 12 consecutive lessons. Within this framework, the theoretical lessons for the experimental group were scheduled in pairs at the beginning of each learning unit, ensuring that students acquired the theoretical foundations before their practical application.

For example, in the learning unit dedicated to endurance running, which consisted of eight lessons in total, the first two sessions were conducted in the classroom and focused on introducing theoretical concepts related to aerobic capacity—its definition, physiological mechanisms, methods of development, and applicability in endurance effort. The subsequent six lessons were conducted on the field, where students applied and practiced the practical components of this topic. This introductory structuring logic was applied consistently across all learning units, ensuring pedagogical coherence between the theoretical and practical components.

Both groups (control and experimental) were assessed before and after the intervention using the instrument previously developed.

### 2.3 The focus-group interview

To investigate students' experiences with the use of the Kahoot platform during the educational intervention, a focus-group interview was conducted with the students in the experimental group immediately after the intervention was completed. The purpose of this qualitative component was to address the research question: How did students perceive the learning experience facilitated through the Kahoot platform during the intervention?

In relation to this question, several specific objectives were pursued: identifying the aspects students found attractive or motivating when using Kahoot; exploring the difficulties they encountered during the activities; evaluating their perceptions of the platform's educational usefulness; and capturing any changes in their engagement and interest in learning.

All students in the experimental group participated in the discussion, which was moderated by the researcher and assisted by an observer who documented nonverbal elements and interaction dynamics. Before the interview began, the moderator conducted a brief introduction outlining the purpose of the research and the interview procedure, while also clarifying that all information collected would remain confidential and anonymized in the final report. The audio recording of the session was initiated only after written parental consent had been obtained, and students were informed that they could refuse to answer any question or withdraw from the discussion at any time without any consequences.

The moderator also established and presented the participation rules to maintain an orderly dynamic and balanced involvement: the moderator asks the questions, students raise their hands to request the floor, and contributions are made only after they are invited to speak. Additional rules regarding respect for differing opinions and the avoidance of interruptions were explicitly stated to create a safe, non-judgmental environment conducive to open expression.

The discussion was guided by a set of open-ended questions included in a semi-structured interview guide; however, the moderator allowed flexibility to encourage the emergence of themes relevant from the students' perspective. No fixed duration was imposed—the interview continued until the topic reached natural saturation, at which point no new information emerged and participant responses became redundant.

The entire focus group was audio recorded and transcribed verbatim. The data were then analyzed using thematic analysis, following the steps outlined by Braun and Clarke (36, 37): (1) familiarizing with the data; (2) generating initial codes; (3) searching for themes; (4) reviewing themes; (5) describing the themes; and (6) producing the report. The entire process adhered to ethical principles of educational research, ensuring informed consent, confidentiality, and the right to withdraw.

### 2.4 Data analysis

The quantitative and qualitative data were analyzed separately using appropriate methods and then integrated during the interpretation phase, following a mixed-methods complementary design. Quantitative data were analyzed using statistical tests to evaluate the reliability of the instrument and the effectiveness of the intervention, while qualitative data from the focus-group interview were analyzed using thematic analysis. The integration of findings occurred at the interpretation stage, where qualitative results were used to complement and provide explanatory context for the quantitative outcomes, particularly in relation to students' learning experiences and engagement. This approach allowed for a more comprehensive understanding of both the effectiveness of the intervention and the educational relevance of the instrument.

Statistical analyses were conducted using the numiqo online statistical platform (38). Descriptive statistics (means and standard deviations) were calculated, and the significance level was set at *p* < .05 (two-tailed). The assumptions of normality and homogeneity of variances were assessed using the Shapiro–Wilk, and Levene's tests.

Independent samples t-tests were used to examine differences between the experimental and control groups, including baseline equivalence, while paired samples t-tests were used to assess changes between pre-test and post-test within each group. Effect sizes were calculated using Cohen's d.

## 3 Results

As in the Methods section, the research findings are reported across three subsections, corresponding to the following components: the development of the instrument, the results of the intervention, and the outcomes of the focus-group interview.

### 3.1 Assessment instrument

*Stage 1.* After quantifying the number of pages allocated to each chapter in the Romanian PE textbooks, the corresponding percentage of items assigned to each factor was calculated as follows: Organization of Motor Activities—4 items (16%); Harmonious Physical Development—8 items (32%); Motor Skills—6 items (24%); Sports Disciplines—3 items (12%); Hygiene and Individual Protection—2 items (8%); Behaviors and Attitudes—2 items (8%).

*Stage 2.* In this stage, a total of 831 items were generated according to the established criteria. Of these, 81 items were removed due to similarity.

*Stage 3.* Cronbach's alpha was calculated for the four scoring dimensions. The results indicated an alpha of 0.945 for the first dimension, 0.911 for the second, 0.871 for the third, and 0.956 for the fourth. All values exceeded the *α* > 0.8 threshold, confirming strong internal consistency and validating the assessment panel.

Following the filtering of items according to the methodological criteria of clarity, relevance, and difficulty, version 1.0 of the instrument was obtained.

*Stage 4.* No major issues related to test administration were identified at this stage.

*Stage 5.* The concepts that posed the greatest difficulties for students were “*locomotion motor skill”* (misunderstood by all six students with the lowest scores and by one of the six students with the highest scores) and “*motor capacity*” (frequency: 6 and 2, respectively). It is important to note that the translation of these terms from Romanian may contribute to their slight lack of clarity. However, based on these results, it was concluded that the misunderstanding of these terms stemmed from a low level of prior knowledge rather than from unclear item wording.

*Stage 6.* The paired t-test results [*t*(30) = −5.375, *p* < 0.001] indicated no significant differences between the two sets of scores. A strong positive correlation was found between the test and retest results [*r*(30) = 0.9404, *p* < 0.05], confirming the consistency of the instrument across repeated administrations.

*Stage 7.* The guide for administering the test was fully developed and prepared for use (see Supplementary Materials).

### 3.2 Results of the intervention

The assumption of normality was confirmed using the Shapiro–Wilk test for both groups at pre-test (control: W = 0.95, *p* = 0.33; experimental: W = 0.99, *p* = 0.97) and post-test (control: W = 0.96, *p* = 0.48; experimental: W = 0.95, *p* = 0.27). Homogeneity of variances was also met at pre-test (Levene's *p* = 0.20) and post-test (Levene's *p* = 0.38). An independent samples t-test indicated no significant difference between the groups at baseline [*t*(45) = 0.33, *p* = 0.74], confirming their initial equivalence.

The analysis of the results reveals clear differences between the two groups over the course of the intervention. In the experimental group, K&U test scores increased significantly from pre-test to post-test, as indicated by the paired samples *t*-test [*t*(25) = −8.66, *p* < 0.001], with a large effect size (*d* = 1.7).

In the control group, no significant change was observed [*t*(20) = −0.48, *p* = 0.63], suggesting stability in K&U scores before and after the intervention period.

A comparison of post-test scores between the two groups confirms what is already evident from the results reported above: students in the experimental group achieved significantly higher scores than those in the control group [*t*(45) = −4.75, *p* < 0.001], with a substantial effect size (*d* = 1.39).

Overall, these findings provide an exploratory picture of students' progression throughout the intervention and indicate a clear differentiation between the groups with respect to K&U scores.

### 3.3 Results of the focus-group interview

The analysis of the qualitative data revealed four main themes regarding students' experiences with the use of the Kahoot platform during the theoretical lessons (Table 1).

**Table 1:** Focus-group interview results.

Theme	Description	Data extracts
Enthusiasm and increased involvement	Most students described the Kahoot! experience as fun and motivating	“*I didn't feel like I was learning, it was like a game.*” *(FG1_P2)*
		“*I usually want to play football, but Kahoot! is also cool, especially when I was in first place.*” *(FG1_P9)*
Positive competition	The students perceived the ranking from the Kahoot! as a stimulus	“*I liked to see who was first, but even if I was in 5th place I was happy.*” *(FG2_P3)*
		“*We were competing, but no offense.*” *(FG1_P6)*
Easier learning	Participants felt that the theoretical information was easier to retain through repetition and interactive format	“*I still remember the questions about the rules, because I kept repeating them in the game.*” *(FG1_P1)*
		“*When I answered incorrectly, I would remember it the next time.*” *(FG2_P12)*
Recommendations for improvement	Although appreciated, the method could be varied more often and combined with physical activities	“*I would like there to be games with movement, not just on the phone.*” *(FG2_P9)*
		“*There should be more funny pictures in the questions.*” *(FG1_P4)*

The first theme was increased enthusiasm and engagement, with students describing the activities as enjoyable, motivating, and similar to a game. The second theme concerned positive competitiveness, as participants perceived the Kahoot leaderboards as a stimulating element that did not generate tension among classmates. A third theme related to facilitated learning, with students noting that the repetition of questions and the interactive format helped them retain theoretical information more easily. Finally, a fourth theme emerged regarding suggestions for improvement, with some students proposing to diversify the activities by integrating additional visual elements or incorporating games that involve physical movement.

## 4 Discussions

Owing to the rigorous validation process, the instrument developed in this study can be characterized as comprising items and response options with a high level of clarity, assessing relevant content, and demonstrating a balanced distribution of item difficulty. Furthermore, the reliability of the instrument was confirmed, indicating that the test can yield consistent results across repeated administrations.

This makes not only the instrument itself a valuable outcome of the research, but also the methodology through which it was developed. While the test presented in this paper is standardized for 5th-grade PE content and students in Romania, the development methodology can be successfully applied to create other assessments based on different content areas—including content from entirely different school subjects.

Regarding the results of the educational intervention, the fact that the experimental group accumulated theoretical knowledge is not necessarily surprising, nor is the significant difference observed between the experimental and control groups at the final assessment. However, these large effect sizes should be interpreted cautiously and within the specific context of this sample and intervention. What is noteworthy, however, is that the control group showed no change over the course of an entire school year in terms of theoretical knowledge levels. According to Ennis (39), the absence of any improvement suggests that no real educational process took place. An educational process—particularly one aligned with a PL-oriented curriculum—should lead to gains in both *declarative knowledge* (what students know) and *aplicative knowledge* (what they can do).

This situation in Romanian PE classes is consistent with previous research placing Romania at the bottom of the rankings in terms of PL implementation (1, 40). Although teachers report that they teach theoretical knowledge (41, 42), this appears to remain largely at the declarative level. Romania continues to be a context in which PE lessons rely predominantly on a sport-based approach, in which fitness tests represent a central component (43, 44).

Another important aspect of this paper is the use of Kahoot in PE lessons. This approach proved to be effective and was received with enthusiasm by the students. This suggests that the method can serve as a useful way to deliver the theoretical content required by students (45) in an interactive manner. Moreover, Romanian teachers appear to be open to integrating digital applications into PE instruction (46).

These findings are consistent with previous research on conceptual PE, which has shown that structured theoretical instruction can significantly improve students' knowledge of physical activity concepts when cognitive content is systematically integrated into PE lessons (16, 17). Similarly, research on gamification in PE indicates that game-based digital strategies can enhance student engagement, motivation, and learning outcomes by creating interactive and feedback-rich environments that support cognitive processing (24, 47). The present study extends this body of evidence by demonstrating that a structured Kahoot-supported intervention can effectively enhance the K&U domain of PL within a school-based PE setting.

The findings of this study align with the views of other researchers who emphasize that PE lessons should include a balanced mix of theoretical and practical components (19, 39), and that the K&U domain plays a fundamental role by catalyzing the development of the other domains of PL (12). Furthermore, the positive experience students reported when using Kahoot suggests that this approach may effectively support progress along the PL journey, as conceptualized by Whitehead (13).

The implications of this research operate at two levels. On the one hand, the study contributes to the field of research through both the validation methodology and the assessment instrument itself. On the other hand, the major implications are pedagogical, particularly for PE instruction. The introduction of theoretical lessons at the beginning of each learning unit, combined with the use of digital applications, should attract the interest of PE teachers and those involved in preparing future educators. The minimal resources required for implementing such an approach further strengthen its feasibility.

## 5 Conclusions

This study achieved its two main objectives. First, the research successfully developed and validated a standardized instrument for assessing the K&U domain of PL in PE context. The instrument demonstrated strong content validity, clarity, balanced item difficulty, and excellent test–retest reliability, supporting its suitability for educational and research applications. Second, the results of the quasi-experimental intervention showed that the integration of Kahoot-supported theoretical lessons significantly improved students' K&U scores, while no significant changes were observed in the control group. These findings confirm the effectiveness of structured, digitally supported theoretical instruction in enhancing the cognitive domain of PL in the PE classes.

This research offers a proposal for the initial steps that an Eastern European country such as Romania—still deeply rooted in a sport-based approach to PE—can take toward implementing PL. The solution we put forward highlights the need for PE lessons to incorporate a theoretical component. Such theoretical content plays a key role in fostering motivation, confidence, and physical competence by building a rational and meaningful understanding of the PL journey that students are expected to progress through. In addition, the positive experience students reported with the use of Kahoot indicates that this digital approach can provide enjoyable learning moments, which aligns closely with the philosophy of PL.

The present study provides a practical pathway that begins with the development, validation, and standardization of an instrument for measuring students' theoretical knowledge in PE, and continues by demonstrating the effectiveness of using Kahoot in PE lessons to teach theoretical content in an interactive and engaging manner.

The originality of this research lies in its proposal of a standardized methodology for measuring the theoretical knowledge in Romanian PE discipline, as well as in being the first study to introduce the use of Kahoot in PE lessons within an Eastern European context. This makes the findings relevant for other countries with a strong tradition of sport-based PE, offering guidance to researchers in countries that are moving toward PE practices informed by the principles of PL.

## 6 Limitations

This study has several limitations that should be acknowledged. First, the relatively small sample size and the use of convenience sample limit the generalizability of the findings. Although the results provide meaningful insights into the effectiveness of the intervention, replication with larger and more diverse samples is necessary to strengthen external validity.

Second, the absence of random assignments represents a limitation inherent to the quasi-experimental design. Participants were allocated based on pre-existing class structures, which may introduce potential selection bias and limit the ability to fully attribute observed differences exclusively to the intervention.

Third, the study was conducted in a single school and involved only one teacher, which may restrict the transferability of the findings to other educational contexts. Differences in teaching style, institutional culture, and student characteristics could influence the effectiveness of similar interventions.

Finally, the study focused exclusively on the K&U domain of PL. Given the multidimensional and holistic nature of the PL concept, future research should examine how similar interventions may influence other domains, including motivation, confidence, and physical competence.

## Data Availability

The raw data supporting the conclusions of this article will be made available by the authors, without undue reservation.
