# Supplementary material for: Enhancing the knowledge and understanding domain of physical literacy: instrument validation and a Kahoot-supported intervention in physical education
Source: Front Sports Act Living. 2026 Mar 27;8:1799871. doi: 10.3389/fspor.2026.1799871 (PMC13066270; doi:10.3389/fspor.2026.1799871)
Supplement: Supplementary file 1 [file Table1.docx]

Supplementary Material

# Scoring Grid for Items by the Expert Panel

| **Dimensions** | **Example** | **Response options and assigned values** |
| --- | --- | --- |
| (1) the degree of clarity of the question | Item no. 121: Which of the following terms refers to a deficient segmental or postural attitude? | 0p. cannot understand the question at all |
|  |  | 1p. can only understand some words but not the question |
|  |  | 2p. can understand all the words but not the meaning of the question |
|  |  | 3p. can partially or fully understand the words, but the meaning is different from the intended one |
|  |  | 4p. partially understands the words but guesses the meaning |
|  |  | 5p. understands both the words and the meaning of the question |
| (2) the degree of clarity of the correct answer options | Correct answer options for item no. 71: eating vegetables and fruits / washing my hands frequently | 0p. cannot understand any of the answer options |
|  |  | 1p. understands only some words but not the meaning of the options |
|  |  | 2p. understands all the words but not the meaning of the options |
|  |  | 3p. understands all the words, but the understood meaning is different from the intended one |
|  |  | 4p. partially understands the words but guesses the meaning of the options |
|  |  | 5p. understands both the words and the meaning of the options |
| (3) the degree of difficulty of the item – considering the question and the answer options | Item no. 23: When the teacher gives the command "Attention," what is the position of the arms? (Options: arms crossed over the chest / arms stretched up / arms back with a straight back / arms stretched forward / arms extended alongside the body with palms against the thighs / I don't know) | 0p. the item is very difficult. A 5th-grade student has no knowledge of the subject |
|  |  | 1p. the item is difficult. A 5th-grade student will guess the correct answer |
|  |  | 2p. the item is medium. Less than half of the 5th-grade students can answer correctly |
|  |  | 3p. the item is medium. More than half of the 5th-grade students can answer correctly |
|  |  | 4p. the item is easy. A 5th-grade student can answer correctly |
|  |  | 5p. the item is very easy. All students know the answer |
| (4) the degree of relevance of the concept evaluated within the item | Item no. 72: Select the terms that denote functional indicators. (Correct answer: respiratory rate / heart rate) | 0p. the item is not important at all. Irrelevant information |
|  |  | 1p. the item is slightly important. The information is only for those interested in the subject |
|  |  | 2p. the item is sufficiently important. The information is for general knowledge – less than half would want to know the answer |
|  |  | 3p. the item is sufficiently important. The information is for general knowledge – but more than half would want to know the answer |
|  |  | 4p. the item is important. Necessary information |
|  |  | 5p. the item is very important. The information is extremely important |

# Item Scoring Grid Used in Semi-Structured Interviews

| **The dimension** | **Helpful questions** | **The scores and selection options of the interviewer** |
| --- | --- | --- |
| (1) The degree of understanding of the question | Is there any word you don’t know?  What does the word "X" mean?  What do you understand when reading this question? | 0p. Failed to understand the question at all; 1p. Understands only some words but not the question; 2p. Understands all the words but not the meaning; 3p. Understands the words partially or fully, but the meaning is different from the intended one; 4p. Partially understands the words but guesses the meaning; 5p. Understands both the words and the meaning of the question. |
| (2) The degree of understanding of the correct answer options | Is there any word you don’t know?  What does the word "X" mean?  What do you understand when reading the answer options? | 0p. Failed to understand any of the answer options at all; 1p. Understands only some words but does not understand the meaning of the options; 2p. Understands all the words but does not understand the meaning of the options; 3p. Understands all the words, but the meaning is different from the intended one; 4p. Partially understands the words but guesses the meaning of the options; 5p. Understands both the words and the meaning of the options. |

# Application Guide

| **General Overview**  This document is an annex to the test designed to measure the K&U level of 5^th^-grade students in the discipline of PE, providing the necessary information that a teacher or any other person administering the test must know.  The objective of the measurement instrument is to collect data that reflects the students’ K&U level in PE, based on specialized documents and/or textbooks.  The test may be administered by the teacher or by any person who reads and understands this material.  The test can be administered in both traditional format (printed on paper) and electronically using platforms such as Google Forms.  The test contains 25 items (questions) to which students must respond without receiving additional support or external guidance.  The instruction text must be read to the students before the test begins, and from the moment the test starts (when students receive it), they have 25 minutes available. |
| --- |
| **Instruction Text to Be Read to Students Before the Test:**  “This test is intended to support the evaluation of your theoretical knowledge in the discipline of PE.  From the moment you receive the test, you have 25 minutes available. This means one minute for each of the 25 questions.  The questions are multiple-choice, with 6 answer options, one of which is ‘I don’t know’.  If there are questions for which you do not know the answer, or you do not understand certain terms used, please select the answer option ‘I don’t know’.  Each question may have one correct answer or multiple correct answers.  Answer all the questions.  Good luck!” |
| **Instructions for Collecting and Interpreting Results**  For each correct answer, the student receives 4 points.  This means that a student answering all 25 questions correctly receives 100 points — equivalent to the grade 10.  If a question has multiple correct answer options and the student does not select all of them, the score for that question is 0 points.  In other words, the student must select all correct answers in order to receive the 4 points. |

# The test

The test included in the supplementary materials has been intentionally preserved in its original Romanian formulation. This decision is essential because the test was standardized and validated within the Romanian educational context, using linguistic structures, terminology, and conceptual formulations specific to the national curriculum for Physical Education and Sports.

Maintaining the original Romanian wording ensures the integrity, reliability, and comparability of the instrument. Any translation or adaptation could potentially alter the meaning of certain items, the nuance of the terms, or the cognitive demands placed on students, which would compromise the standardized nature of the test and its psychometric properties.

For these reasons, the Romanian version included here reflects the authentic format used during validation and application, preserving its educational and scientific accuracy.

| **no.** | **Test Item** | **Response Options** | **Corect**  **answer(s)** |
| --- | --- | --- | --- |
| 1 | Care dintre următorii termeni desemnează segmente ale corpului uman? | cap / inimă / coapsă / biceps / triceps / nu știu | cap / coapsă |
| 2 | Ce acțiune efectuezi atunci când profesorul dă următoarea comandă: „La dreapta!”? | pornesc în mers către dreapta/ pornesc în alergare către dreapta / întorc capul către colegul din dreapta / pun mâna dreapta pe șold și mă alineez la dreapta / mă întorc la dreapta / nu știu | mă întorc la dreapta |
| 3 | Colectivul clasei tale se află în formația “în linie pe un rând” și au efectuat numărătoarea “câte doi”, iar ție ți-a fost repartizat numărul 2. Ce acțiune efectuezi când profesorul dă următoarea comandă: “Din linie pe un rând în linie pe două rânduri!”? | formez o pereche cu un elev care are numărul 1/ mă așez față în față cu un elev care are numărul 1/ fac un pas înapoi situându-mă înapoia elevilor cu numărul 1 / fac un pas oblic spre înainte dreapta, așezându-mă în fața elevilor cu numărul 1 / ne deplasăm câte doi de mână apucat / nu știu | fac un pas oblic spre înainte dreapta, așezându‑mă în fața elevilor cu numărul 1 |
| 4 | Atunci când profesorul dă comanda “Drepți!”, care este poziția brațelor? | brațele sunt încrucișate la piept / brațele sunt întinse sus / brațele înapoi cu spatele drept / brațele întinse înainte / brațele întinse pe lângă corp cu palmele lipite de coapse / nu știu | brațele întinse pe lângă corp cu palmele lipite de coapse |
| 5 | Capul este un segment al corpului. Ce mișcări pot fi realizate cu acest segment? | aplecare / forfecare / coborâre / răsucire / extensie / nu știu | aplecare / răsucire / extensie |
| 6 | Trunchiul este un segment al corpului. Ce mișcări pot fi realizate cu acest segment? | îndoire / îndoire răsucită / înclinare / forfecare / coborâre / nu știu | îndoire / îndoire răsucită / înclinare |
| 7 | Profesorul dă comanda “Drepți!”. În ce poziție trebuie să fie călcâiele după această comandă? | călcâiele sunt ușor depărtate cu vârfurile picioarelor lipite / câlcâiele sunt ușor ridicate de pe sol / câlcâiele sunt lipite cu vârfurile ușor depărtate / călcâiul stâng este pe sol și cel drept este ușor ridicat / călcâiul drept este pe sol și cel stâng este ușor ridicat / nu știu | călcâiele sunt lipite cu vârfurile ușor depărtate |
| 8 | Ce măsuri poți lua singur pentru a menține starea de sănătate a organismului? | să consum legume și fructe / să nu transpir / să mă spăl des pe mâini / să iau antibiotice dacă sunt răcit / să nu alerg / nu știu | să consum legume și fructe / să mă spăl des pe mâini |
| 9 | Care dintre următorii termeni desemnează poziții de bază ale corpului uman? | în coloană câte unu / culcat facial / în linie pe două rânduri / atârnat / în cerc de mâini apucat / nu știu | culcat facial / atârnat |
| 10 | Care dintre următorii termeni desemnează mișcări de bază ale segmentelor corpului uman? | contracția musculară / inspirația / expirația / răsucirea trunchiului / relaxarea musculară / nu știu | răsucirea trunchiului |
| 11 | Ce se întâmplă cu frecvența respiratorie atunci când depunem efort fizic? | respirația devine mai profundă / respirația se blochează / respirația se răcește / frecvența respiratorie scade / frecvența respiratorie crește / nu știu | frecvența respiratorie crește |
| 12 | Ce este frecvența cardiacă? | numărul de respirații într-un minut / o boală a plămânilor / o boală a inimii / numărul de bătăi ale inimii într-un minut / viteza de deplasare / nu știu | numărul de bătăi ale inimii într-un minut |
| 13 | În ce ordine se execută complexul de exerciții de dezvoltare fizică armonioasă? | se poate executa în orice ordine / se execută în funcție de necesități / se execută în ordinea: membre inferioare – trunchi – membre superioare – cap / se execută în ordinea: cap – membre superioare – trunchi – membre inferioare / se execută mai întâi exercițiile de îndemânare și apoi cele de forță / nu știu | se execută în ordinea: cap – membre superioare – trunchi – membre inferioare |
| 14 | Care sunt cele două componente ale capacității motrice? | frecvența respiratorie și frecvența cardiacă / înălțimea și greutatea / încălzirea și revenirea după efort / oxigenul și dioxidul de carbon / calitățile motrice și deprinderile motrice / nu știu | calitățile motrice și deprinderile motrice |
| 15 | Care din următorii termeni desemnează o deprindere motrică? | viteza/ îndemânarea / alergarea / rezistența / mersul / nu știu | alergarea / mersul |
| 16 | Care din următorii termeni desemnează o calitate motrică? | forța / talia / perimetrul abdominal / anvergura brațelor / indicele de masă corporală / nu știu | forța |
| 17 | Care din următorii termeni desemnează o deprindere motrică de locomoție de bază? | ștul la poartă / săritura / alergarea / pasul de sus cu două mâini / îndemânarea / nu știu | săritura / alergarea |
| 18 | Ce este escaladarea? | o activitate interzisă copiilor / o calitate motrică / o stare de spirit / o deficiență posturală / o deprindere motrică / nu știu | o deprindere motrică |
| 19 | Calitatea motrică “îndemânarea” te ajută să: | îți dezvolți echilibrul / te orientezi mai bine în spațiu / îți coordonezi corpul și segmentele acestuia / execuți cu ușurniță și precizie mișcări dificile / manevrezi obiectele cu ușurință / nu știu | îți dezvolți echilibrul / te orientezi mai bine în spațiu / îți coordonezi corpul și segmentele acestuia / execuți cu ușurință și precizie mișcări dificile / manevrezi obiecte cu ușurință |
| 20 | Cărui joc sportiv îi aparține proba “săritura în lungime cu elan”? | fotbal / handbal / sărituri în apă / atletism / nu este o probă sportivă / nu știu | atletism |
| 21 | Câți jucători conține o echipă de handbal? | 6 jucători de câmp și un portar / 5 jucători de câmp și un portar / 7 jucători de câmp și un portar / 8 jucători de câmp și un portar / 10 jucători de câmp și un portar / nu știu | 6 jucători de câmp și un portar |
| 22 | Cărui joc sportiv îi aparține procedeul tehnic “serviciu de jos din față”? | fotbal / rugby / șah / baschet / volei / nu știu | volei |
| 23 | Care din următoarele exerciții pot fi utilizate pentru pregătirea organismului pentru efort (încălzire)? | șuturi la poartă / săritura peste lada de gimnastică / mers pe vârfuri / mers cu pas fandat / alergare cu genunchii sus / nu știu | mers pe vârfuri / mers cu pas fandat / alergare cu genunchii sus |
| 24 | Ce trebuie să faci în cazul în care resimți o stare de vomă în timpul lecției de educație fizică și sport? | nu te mai gândești la asta și va trece / respiri adânc până simți o ușoară amețeală / anunți un coleg și continui activitatea / îți iei telefonul mobil și îți suni părinții de urgență / te oprești din efort și anunți profesorul / nu știu | te oprești din efort și anunți profesorul |
| 25 | Care dintre următoarele comportamente sunt recomandate atunci când echipa ta a pierdut la un joc? | felicitarea adversarilor / găsirea vinovatului pentru înfrângere / certarea arbitrului dacă acesta a greșit împotriva echipei tale / mulțumești coechipierilor pentru joc / propunerea unui alt joc la care crezi că vei putea câștiga / nu știu | felicitarea adversarilor / mulțumești coechipierilor pentru joc |
